# Assessing Surgical Outcomes in Cervical Degenerative Disease: The Role of Intraoperative Neurophysiological Monitoring

**DOI:** 10.3390/jcm14113771

**Published:** 2025-05-28

**Authors:** Delia Cannizzaro, Carlo Cossa, Giovanni Marco Sicuri, Matteo Riccardo Minotti, Lucia Politini, Jad El Choueiri, Francesca Matteo, Angelo Rusconi, Roberto Stefini

**Affiliations:** 1 Department of Neurosurgery, ASST Ovest Milano Legnano Hospital, 20025 Legnano, Milan, Italy; delia.cannizzaro@asst-ovestmi.it (D.C.);; 2 Humanitas University, Via Rita Levi Montalcini 4, 20072 Pieve Emanuele, Milan, Italy; 3 Department of Neurology, ASST Ovest Milano Legnano Hospital, 20025 Legnano, Milan, Italy; 4 Faculty of Medicine and Surgery, University of Roma Tor Vergata, 00133 Rome, Italy

**Keywords:** intraoperative neuromonitoring, cervical degenerative disease, myelopathy, spine surgery predictors, long-term neurological outcomes

## Abstract

**Background:** Cervical degenerative disease is a common condition associated with significant morbidity, often presenting as neck pain, radiculopathy, or myelopathy. Its growing incidence, particularly in the aging population, has led to an increased demand for surgical interventions aimed at relieving neural compression and restoring spinal stability. **Objective**: This study aims to evaluate surgical outcomes in patients with degenerative cervical conditions, with a particular focus on the role of intraoperative neurophysiological monitoring (IONM) in preventing adverse neurological events both immediately postoperatively and at long-term follow-up. **Methods**: A retrospective analysis was performed on patients who underwent cervical spine surgery for degenerative conditions between January 2021 and June 2024. Data collected included demographics, comorbidities, surgical details, and intraoperative neurophysiological monitoring. Surgical outcomes were assessed using the modified Rankin Scale (mRS), Odom’s Criteria, and the modified Japanese Association (mJOA) score. **Results**: Key findings demonstrated that advanced age and the presence of preoperative myelopathy were significantly associated with poorer postoperative outcomes across all evaluated measures. Conversely, factors such as gender, surgical approach, and the number of treated levels did not significantly influence recovery. Although intraoperative neurophysiological monitoring (IONM) did not show an immediate effect on postoperative outcomes, it was linked to prognostic value for long-term neurological status, suggesting a potential protective role in preserving neurological function. **Conclusions:** This study identifies age, preoperative functional status, and myelopathy as crucial predictors of postoperative recovery in cervical spine surgery for degenerative disease. These findings underscore the importance of early intervention in patients with myelopathy and highlight the complex role of IONM in improving long-term neurological outcomes. IONM changes may help identify patients at higher risk of poor recovery who could benefit from intensive postoperative rehabilitation. Further prospective studies are warranted to elucidate the complex interactions between patient characteristics and surgical factors in optimizing postoperative recovery.

## 1. Introduction

Cervical degenerative disease (CDD) is a prevalent condition affecting the cervical spine, often resulting in significant morbidity characterized by neck pain, radiculopathy, and myelopathy [1]. Degenerative alterations involving the vertebrae, intervertebral disks, facet joints, and ligaments form the pathological basis of cervical spondylosis [2]. These structural changes can cause cervical spondylotic myelopathy (CSM) through direct compression of the spinal cord and/or adjacent vascular structures [3].

With the aging population, the incidence of degenerative cervical conditions is steadily increasing, leading to a growing demand for surgical intervention in advanced cases [4]. Surgical management—often involving decompression and anterior or posterior fusion—is frequently necessary to alleviate neural compression and restore spinal stability in patients who do not respond to conservative treatments [2]. Surgical outcomes for cervical degenerative disease (CDD) can be influenced by multiple factors, including patient age, severity of degeneration, surgical approach, and the presence of preoperative neurological deficits. Myelopathy, in particular, is a critical prognostic indicator, as patients exhibiting clinical and radiological evidence of spinal cord involvement generally experience poorer postoperative outcomes [5]. Consequently, identifying the key determinants of surgical success and neurological recovery is vital to optimize patient care and enhance long-term results [6]. In this context, intraoperative neurophysiological monitoring (IONM) has emerged as an important adjunct during cervical spine surgery [7,8]. Intraoperative neurophysiological monitoring (IONM) provides real-time feedback on spinal cord and nerve root function, enabling surgeons to detect early signs of neurological compromise during surgery and implement immediate corrective measures, thereby potentially preventing permanent deficits. Although IONM use is well established in spinal tumor surgery, where it has become standard practice, its application in degenerative spinal diseases remains controversial, with conflicting evidence regarding its efficacy in improving postoperative neurological outcomes [9,10].

The differences observed among study groups regarding the outcomes of neurophysiological monitoring in degenerative spinal surgery represent a strong motivation for conducting this study. Various authors report differing analyses concerning the use of IONM [7,8,10], emphasizing the need for further investigation. This study aims, through a large surgical case series, to lay the groundwork for future in-depth research, where the application of neurophysiological techniques and the evaluation of their responses can be standardized.

To explore the potential role of intraoperative neurophysiological monitoring (IONM) in degenerative spinal pathology, we conducted a retrospective analysis of a surgical series involving patients with degenerative cervical disease, focusing specifically on IONM’s impact. Immediate and long-term neurological outcomes were assessed using the modified Rankin Scale (mRS), modified Japanese Orthopaedic Association (mJOA) score, and Odom score. By examining the influence of patient characteristics, surgical variables, and IONM use on postoperative recovery, this study aims to identify key predictors of surgical success in managing cervical degenerative disease.

In summary, this study seeks to extend the well-established application of IONM from spinal tumor surgery to cervical degenerative pathology—a condition of increasing prevalence due to rising life expectancy and a growing challenge in neurosurgical practice.

## 2. Materials and Methods

### 2.1. Study Design

A retrospective chart review was conducted to collect clinical and surgical data from 100 patients who underwent surgical treatment for degenerative cervical spine conditions between January 2021 and June 2024 at the Department of Neurosurgery, Ospedale Nuovo di Legnano. The study aimed to evaluate surgical outcomes using established clinical metrics, including the modified Rankin Scale (mRS), Odom’s Criteria (ODAM) score [9,11], and the modified Japanese Orthopaedic Association (mJOA) score [12]. Immediate postoperative and long-term neurological outcomes were analyzed in relation to patient age, surgical approach, number of levels treated, preoperative neurological status, and the presence of clinical and radiological myelopathy. Additionally, the study assessed outcomes concerning intraoperative neurophysiological monitoring (IONM) changes compared to baseline values.

### 2.2. Patient Population

Patients included in this study were those diagnosed with degenerative cervical spine pathologies such as cervical spondylotic myelopathy, cervical disk herniation, and cervical stenosis. Only cases involving the C3 to C7 level were studied. Inclusion criteria were as follows: Age ≥ 18 years, documented degenerative cervical spine disease confirmed by MRI/CT scans, completion of a minimum follow-up period of 30 days, availability of preoperative and postoperative clinical data for mRS, ODAM, and mJOA scores. Patients with trauma, infections, tumors, or previous cervical spine surgery were excluded.

### 2.3. Surgical Procedure

All patients underwent anterior, posterior, or combined cervical spine surgical approaches, selected according to the severity and location of their degenerative pathology. The choice of approach was determined based on the K-line criteria [13].

### 2.4. Intraoperative Neurophysiological Monitoring

All patients included in this study underwent surgical procedures with the assistance of IONM. Our neurophysiological protocol utilized electromyography (EMG), somatosensory evoked potentials (SSEPs), and motor evoked potentials (MEPs) to provide real-time assessment of the functional integrity of neural structures during surgery. Changes exceeding 50% from baseline values in these parameters were considered significant. Upon the occurrence of an IONM alert, immediate adjustments were made to both surgical and anesthetic management, including irrigation, alteration of the surgical site, administration of steroid therapy, and elevation of blood pressure [14,15].

### 2.5. Outcome Measures

The primary outcomes were assessed using the following scales: Modified Rankin Scale (mRS): Employed to evaluate the degree of functional disability both preoperatively and at follow-up [11]. Odom’s Criteria: Used to assess clinical success postoperatively, categorized into four grades (Excellent, Good, Fair, Poor) based on patient-reported outcomes and clinical evaluation of symptom improvement [11]. Modified Japanese Orthopaedic Association (mJOA) score: Utilized to assess neurological function, with emphasis on motor and sensory impairments as well as bladder function [12].

### 2.6. Data Collection

Preoperative, immediate postoperative, and long-term follow-up data were extracted from electronic medical records. Follow-up assessments were standardized to the last outpatient visit or, if the patient was clinically well, the last telephone contact. Collected data included patient demographics, comorbidities, surgical details (number of levels treated, surgical approach, presence of clinical or radiological myelopathy), and intraoperative monitoring parameters (significant changes exceeding 50% from baseline).

### 2.7. Statistical Analysis

Statistical analyses were performed using Excel, R Studio, and Python (Excel Microsoft 365 Office, Version 2504. R Studio 2023.03.0+386. Python 3.12.3). Independent t-tests were used to compare continuous variables—such as mRS, ODAM, and mJOA scores—across subgroups defined by age (<60 vs. ≥60), gender (male vs. female), surgical approach (anterior vs. posterior), and number of levels operated (single-level vs. multi-level). Chi-square tests were applied for categorical variables. A *p*-value of less than 0.05 was considered statistically significant for all tests.

## 3. Results

**Figure 1:**
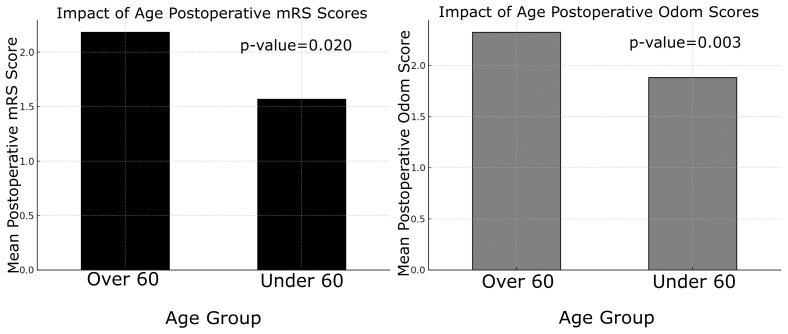
On the left, “Impact of Age on Postoperative mRS Scores” is shown; on the *y* axis, the mean postoperative mRS scores. On the right, the “Impact of Age on Postoperative Odom Scores” is shown, with bars representing different age groups (over and under 60 years).

This retrospective analysis included 100 patients who underwent neurosurgical treatment for degenerative cervical spinal pathology (C3–C7) between January 2021 and June 2024. The cohort comprised 64 males and 36 females, with a mean age of 60.7 years (range: 34.5 to 83 years). (see Table 1) Among these patients, 88 were diagnosed with clinical and radiological myelopathy, while 12 did not present this condition. Surgical interventions were performed via different approaches: 67 anterior, 28 posterior, and 5 combined (anterior + posterior). (see Table 2) To compile our database, patient records were reviewed to extract data from the neurological physical examination, including information on motor function, sensory disturbances, and sphincter control. Additionally, in the postoperative period, besides these elements, the patient’s ability to perform daily activities was also assessed. These factors were evaluated to assess their impact on postoperative neurological outcomes using the modified Rankin Scale (mRS), modified Japanese Orthopaedic Association (mJOA) score, and Odom’s criteria. Age emerged as a significant determinant of outcomes. Patients over 60 years demonstrated worse immediate postoperative mRS (t = 2.37, *p* = 0.020) and Odom scores (t = 3.05, *p* = 0.003) compared to those under 60 (Figure 1). However, no significant differences were found in mJOA scores between the age groups, either postoperatively or at follow-up and no biases were observed due to different genders, thanks to the homogeneity of the database.

**Figure 2:**
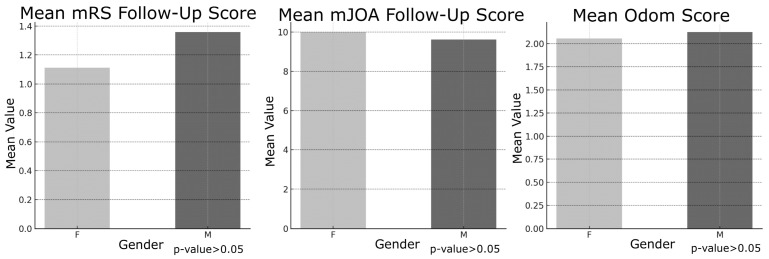
On the left “Mean mRS Follow-up Score” based on Gender (F = Female; M = Male). In the middle “Mean mJOA Follow-up Score” based on Gender (F = Female; M = Male). On the right “Mean Odom Follow-up Score” based on Gender (F = Female; M = Male).

Gender, however, did not significantly influence outcomes, as male and female patients exhibited comparable postoperative mRS, mJOA, and Odom scores (all *p*-values > 0.05; Figure 2).

**Figure 3:**
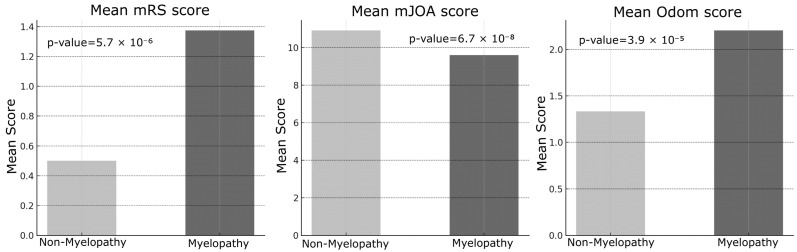
On the left, “Mean mRS Score” based on Myelopathy condition (Non-Myelopathy = absent; Myelopathy = present). In the middle “Mean mJOA Score” based on Myelopathy condition (Non-Myelopathy = absent; Myelopathy = present). On the right, “Mean Odom Score” based on Myelopathy condition (Non-Myelopathy = absent; Myelopathy = present).

Myelopathy was strongly associated with poorer outcomes across all evaluated metrics. Patients with myelopathy demonstrated significantly worse postoperative scores on the mRS (t = 5.85, *p* = 5.7 × 10^−6^), mJOA (t = −7.14, *p* = 6.7 × 10^−8^), and Odom criteria (t = 5.40, *p* = 3.9 × 10^−5^), a pattern that persisted at long-term follow-up (Figure 3).

**Figure 4:**
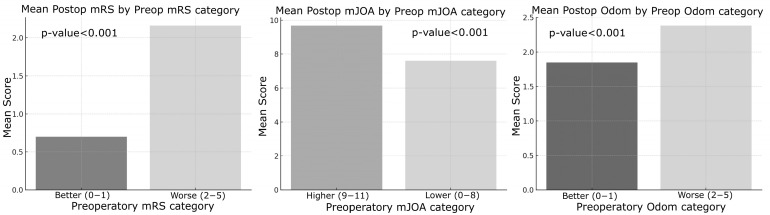
On the left, “Mean Postoperative mRS Score by Preoperative mRS Category” based on Better (0, 1/5 points) and Worse (2, 3, 4, 5/5 points) categorization. In the middle “Mean Postoperative mJOA Score by Preoperative mJOA Category” based on Better (0, 1/5 points) and Worse (2, 3, 4, 5/5 points) categorization. On the right “Mean Postoperative Odom Score by Preoperative Odom Category” based on Better (0, 1/5 points) and Worse (2, 3, 4, 5/5 points) categorization.

Preoperative functional status was a significant predictor of postoperative outcomes. Patients with poorer preoperative mRS scores (2–5) exhibited significantly worse postoperative mRS and mJOA scores (*p* < 0.001). Similarly, patients with lower preoperative mJOA scores (0–8) showed significantly poorer postoperative outcomes across all measured metrics (*p* < 0.001, Figure 4).

**Figure 5:**
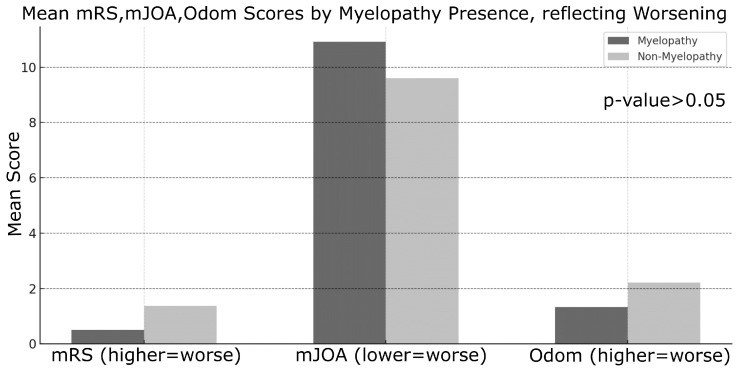
Mean outcome scores by myelopathy presence, reflecting worsening on the left for mRS scores, in the middle for mJOA scores, on the right for Odom scores. Each of the 3 histograms are characterized by 2 columns based on Myelopathy condition (Non-Myelopathy = absent; Myelopathy = present).

Comparison between single-level and multi-level surgeries revealed no significant differences in outcome measures (*p* > 0.05). Similarly, the surgical approach—whether anterior, posterior, or combined, as determined by the K-line—did not significantly influence immediate or long-term outcomes. Nonetheless, patients with myelopathy consistently exhibited poorer results across all assessed metrics (Figure 5).

Intraoperative neurophysiological monitoring (IONM) did not significantly influence immediate postoperative outcomes; however, it was significantly associated with differences in long-term mJOA scores (t = −2.97, *p* = 0.020). Specifically, patients who experienced intraoperative neurophysiological alerts (sensory and/or motor) demonstrated a lower likelihood of long-term neurological recovery compared to those without such alerts. No significant correlations were found between IONM status and either mRS or Odom scores. Although no statistically significant differences were observed regarding the loss or reduction in IONM signals, this may be due to the limited number of alert cases in this cohort. Out of 100 patients, 8 exhibited intraoperative IONM changes, whereas 92 showed no recorded changes.

In summary, age, presence of myelopathy, and preoperative functional status were identified as the strongest predictors of postoperative outcomes. Conversely, surgical approach, gender, and the number of treated levels did not significantly impact recovery. Although intraoperative neurophysiological monitoring showed an association with long-term mJOA scores, it did not significantly influence other outcome measures.

## 4. Discussion

The results of this study provide important insights into the factors influencing surgical outcomes in patients with cervical degenerative disease (CDD). Key findings emphasize the impact of patient demographics, preoperative clinical status, and surgical variables on both immediate and long-term neurological recovery [11]. One of the most notable findings was the influence of age on surgical outcomes. Patients over 60 years of age exhibited significantly worse immediate postoperative mRS and Odom scores compared to younger patients, suggesting that advanced age may be associated with slower initial recovery and poorer functional outcomes. However, no significant differences were observed in mJOA scores postoperatively or at long-term follow-up across age groups. This discrepancy may indicate that, despite greater functional disability, neurological recovery as assessed by mJOA remains comparable regardless of age. The identification of age as a key determinant of surgical outcome aligns with prior studies, which have attributed poorer results in older patients to reduced physiological reserve and the higher prevalence of comorbidities [16]. In contrast, gender did not significantly affect any outcome measures, indicating that male and female patients demonstrate comparable recovery profiles. This observation is consistent with previous studies reporting minimal gender-based differences in surgical outcomes following cervical spine surgery [17,18]. An intriguing finding of this study was the lack of significant differences in outcomes between single-level and multi-level surgeries. Despite the expectation that multi-level procedures might yield poorer results due to increased complexity and longer operative times, our data did not confirm this hypothesis. Similarly, the surgical approach, whether anterior or posterior—did not significantly impact outcome measures, suggesting that both techniques can be equally effective when appropriately selected based on the patient’s specific pathology. These results emphasize the importance of individualized surgical planning tailored to anatomical and disease characteristics rather than relying on a uniform approach. This conclusion aligns with the recent literature indicating that the choice of surgical approach (anterior versus posterior) does not substantially affect clinical outcomes [19,20,21]. However, the association between the number of levels treated and favorable surgical outcomes remains controversial, as it is influenced by multiple factors such as operative time and the severity of preoperative radiological findings. As expected, patients with myelopathy demonstrated significantly worse outcomes compared to those without this condition. Across all assessed metrics—including mRS, mJOA, and Odom scores—individuals with myelopathy exhibited reduced functional and neurological recovery both in the immediate postoperative period and at long-term follow-up. These results highlight the pivotal role of myelopathy as a predictor of poor outcomes, likely reflecting irreversible spinal cord damage occurring prior to surgical intervention [22,23]. Early detection and timely intervention in patients with myelopathy are essential to minimize permanent neurological deficits. Preoperative functional status, as measured by mRS and mJOA scores, showed a strong correlation with postoperative outcomes. Patients presenting with poorer preoperative functional status (i.e., higher mRS or lower mJOA scores) consistently experienced less favorable postoperative recovery [24]. This finding emphasizes the critical importance of baseline neurological status in predicting surgical outcomes, underscoring the need for timely surgical intervention before significant neurological deterioration occurs. Interestingly, the role of intraoperative neurophysiological monitoring (IONM) in this study exhibited a nuanced effect. Although IONM did not significantly impact immediate postoperative outcomes, it showed a significant association with long-term mJOA scores, suggesting a potential protective role against progressive neurological decline over time. Patients who experienced intraoperative neurophysiological alerts during decompressive surgery tended to have reduced long-term neurological improvement compared to those without such changes. For these patients, implementing a focused and structured motor rehabilitation program is strongly recommended. The use of IONM in spinal surgery proves to be cost-effective. As value-based medicine continues to grow and evolve, the need for such evaluations will rise, helping surgeons make informed, sustainable decisions that benefit both patients and the healthcare system [25]. Given these findings, the use of intraoperative neuromonitoring (IONM) in cervical spine surgery, particularly in high-risk patients, remains warranted; however, its precise role requires further elucidation. This perspective is supported by a recent meta-analysis conducted by El Choueiri et al., 2025 [26], However, the meta-analysis found no statistically significant difference in neurological outcomes between patients undergoing surgery for cervical degenerative spine disease with versus without IONM. It emphasized that the decision to utilize IONM should be made by the surgeon on a case-by-case basis and highlighted the need for further research. Intraoperative variations in IONM signals often prompt adjustments in surgical and anesthetic management, including prolonged waiting periods, more cautious maneuvers during alerts, administration of steroids, and blood pressure modulation. These interventions likely explain why IONM alerts do not significantly affect immediate postoperative outcomes, as they serve to prevent or minimize neurological deterioration. The relatively small number of patients experiencing IONM changes limited the statistical analysis differentiating between signal loss and amplitude reduction, as well as the assessment of outcomes related to potential recovery of neurophysiological signals. Nonetheless, intraoperative alerts hold significant prognostic value for long-term neurological recovery, allowing clinicians to identify patients who may benefit from targeted intensive rehabilitation during the postoperative period. Subgroup analyses using Chi-square and ANOVA tests indicated that neither the presence of myelopathy nor the use of intraoperative neurophysiological monitoring (IONM) significantly influenced the likelihood of postoperative improvement in mRS or mJOA scores, contrasting with earlier findings from t-tests. These results highlight the complex and multifactorial nature of surgical outcomes in cervical degenerative disease (CDD), suggesting that, while factors such as age and myelopathy independently predict outcomes, their interactions with other variables may mask broader associations. IONM has become an invaluable tool in predicting long-term neurological outcomes for patients undergoing surgery involving the nervous system. By providing continuous, real-time feedback on neural function, IONM facilitates the early detection of potential intraoperative neural injury, allowing timely corrective interventions. This study demonstrated that changes in IONM signals, including somatosensory evoked potentials (SSEPs) and motor evoked potentials (MEPs)—correlate with long-term neurological outcomes. Furthermore, the maintenance of stable IONM signals throughout surgery is frequently associated with favorable long-term recovery, including improved motor and sensory function. In summary, this study identifies key predictors of surgical outcomes in degenerative cervical spine disease. Age, myelopathy, and preoperative functional status emerged as the most significant determinants of postoperative recovery, whereas surgical approach, number of operated levels, and gender showed no significant impact. The prognostic value of IONM in long-term neurological outcomes appears promising, particularly when integrated with comprehensive clinical assessment and postoperative follow-up. Nevertheless, future prospective studies with larger cohorts and more granular IONM data are needed to further elucidate its role and deepen our understanding of the complex interplay between patient characteristics and surgical factors in CDD management.

## 5. Limitations

Given the retrospective design of this study, potential limitations include selection bias. Additionally, the heterogeneity of surgical techniques and variable follow-up durations among patients may have influenced the outcomes. The involvement of multiple surgeons also introduces procedural variability, which could affect the results. Although the sample size is relatively large compared to similar studies in the literature, multicenter prospective studies are needed to validate these findings. A notable limitation is the small number of intraoperative neurophysiological alerts recorded—only eight cases showed significant changes during surgery. This limited sample size reduces the statistical power of the analysis and may constrain the generalizability of the conclusions. The low incidence of alerts could be attributable to the specific characteristics of the patient cohort, the surgical techniques employed, or the sensitivity thresholds applied during IONM.5. Conclusions. This retrospective study offers meaningful insights into the determinants of surgical outcomes in patients with cervical degenerative disease. Age, preoperative functional status, and the presence of myelopathy were identified as key predictors of postoperative recovery, with older individuals and those with myelopathy experiencing significantly poorer outcomes across multiple clinical measures. Conversely, variables such as gender, surgical approach, and the number of treated levels did not demonstrate a statistically significant association with recovery. Importantly, IONM emerged as a potentially valuable prognostic tool, particularly in its correlation with long-term neurological outcomes as assessed by mJOA scores. These findings highlight the critical role of early diagnosis and timely intervention, especially in patients with myelopathy, to improve postoperative results. IONM changes should help identify patients who may benefit from a more intensive postoperative rehabilitation program, as they appear—based on our results—to have a lower likelihood of functional recovery. Further prospective, large-scale research is necessary to clarify the predictive value of IONM and to better define its role, along with other intraoperative factors, in optimizing surgical outcomes for cervical spinal degenerative disease.

## Figures and Tables

**Table 1:** Clinical characteristics.

Variable	Mean Value
Age at operation	60.72 years
Sex	64 males, 36 females
Time of follow up from op	10.47 months
mRS pre	2.21/6
mJOA pre op	8.17/11
Odom	2.10/4
mRS post	1.87/6
mJOA post	8.71/11
mRS follow up	1.27/6
mJOA follow up	9.76/11

**Table 2:** Surgical characteristics.

Variable	Mean Value
Age at operation	60.72 years
Sex	64 males, 36 females
Single or Multi Levels	Single: 37%, Multi: 63%
Myelopathy	Yes: 88%, No: 12%
Approach	Anterior: 67%, Posterior: 28%, Mixed: 5%
Change in IONM	Yes: 8%, NO: 92%

## Data Availability

The data presented in this study are available on request from the corresponding author. The data are not publicly available due to privacy or ethical restrictions.
